# Proteomic Analysis of Murine Bone Marrow Very Small Embryonic-like Stem Cells at Steady-State Conditions and after In Vivo Stimulation by Nicotinamide and Follicle-Stimulating Factor Reflects their Germ-Lineage Origin and Multi Germ Layer Differentiation Potential

**DOI:** 10.1007/s12015-022-10445-6

**Published:** 2022-08-20

**Authors:** Vira Chumak, Katarzyna Sielatycka, Andrzej Ciechanowicz, Kamila Bujko, Mariusz Z. Ratajczak, Magdalena Kucia

**Affiliations:** 1 Center for Preclinical Studies and Technology, Laboratory of Regenerative Medicine, Medical University of Warsaw, ul. Banacha 1b, Warsaw, Poland; 2 Department of Physiology, Faculty of Biology, University of Szczecin, Felczaka 3c, 71-412 Szczecin, Poland; 3 Department of Physiology, Pomeranian Medical University, 71-252 Szczecin, Poland; 4 Stem Cell Institute at James Graham Brown Cancer Center, University of Louisville, Louisville, KY USA; 5 Centre of Excellence of Medical University of Warsaw for Rare and Undiagnosed Diseases, University of Louisville, Louisville, KY USA

**Keywords:** Nicotinamide, FSH, VSELs, PGCs, Proteomics, Germ line commitment, UN-SDG3

## Abstract

Very small embryonic-like stem cells (VSELs) are a dormant population of development early stem cells deposited in adult tissues that as demonstrated contribute to tissue/organ repair and regeneration. We postulated developmental relationship of these cells to migrating primordial germ cells (PGCs) and explained the quiescent state of these cells by the erasure of differently methylated regions (DMRs) at some of the paternally imprinted genes involved in embryogenesis. Recently, we reported that VSELs began to proliferate and expand in vivo in murine bone marrow (BM) after exposure to nicotinamide (NAM) and selected pituitary and gonadal sex hormones. In the current report, we performed proteomic analysis of VSELs purified from murine bone marrow (BM) after repeated injections of NAM + Follicle-Stimulating Hormone (FSH) that in our previous studies turned out to be an effective combination to expand these cells. By employing the Gene Ontology (GO) resources, we have performed a combination of standard GO annotations (GO-CAM) to produce a network between BM steady-state conditions VSELs (SSC-VSELS) and FSH + NAM expanded VSELs (FSH + NAM VSELs). We have identified several GO biological processes regulating development, organogenesis, gene expression, signal transduction, Wnt signaling, insulin signaling, cytoskeleton organization, cell adhesion, inhibiting apoptosis, responses to extra- and intracellular stimuli, protein transport and stabilization, protein phosphorylation and ubiquitination, DNA repair, immune response, and regulation of circadian rhythm. We report that VSELs express a unique panel of proteins that only partially overlapped with the proteome of BM – derived hematopoietic stem cells (HSCs) and hematopoietic mononuclear cells (MNCs) and respond to FSH + NAM stimulation by expressing proteins involved in the development of all three germ layers. Thus, our current data supports further germ-lineage origin and multi germ layer differentiation potential of these cells.

**Graphical abstract:**

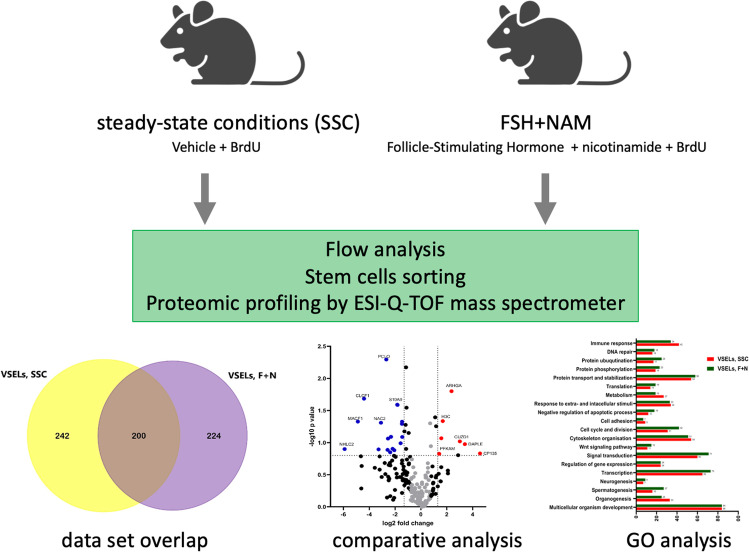

**Supplementary Information:**

The online version contains supplementary material available at 10.1007/s12015-022-10445-6.

## Introduction

Very small embryonic-like stem cells (VSELs) are an intriguing population of stem cells present in adult tissue, including bone marrow (BM) [1]. Identification and purification of these cells by FACS sorter requires a unique gating strategy because of the small size of these cells, which are slightly smaller than erythrocytes [2–4]. Several independent laboratories successfully isolated VSELs from murine BM, peripheral blood, and female and male gonads [5–15]. These cells express some markers characteristic of embryonic stem cells (ESCs) and primordial germ cells (PGCs) and remain quiescent in adult tissues due to the erasure of parental imprinting at differently methylated regions (DMRs) in some genes involved in initiating embryogenesis [16]. In-depth molecular analysis of VSELs has revealed that their quiescence in adult BM is controlled by epigenetic changes to imprinted genes, including the *Igf2-H19* locus, which is erased in murine VSELs similarly as seen in migrating PGCs [17–19]. The epigenetic changes detected in VSELs affect insulin/insulin-like growth factors signaling. We also already reported that VSELs in the presence of nicotinamide (NAM) regain proper somatic methylation on selected DMRs in a mechanism involving the action of de-novo DNA methyltransferases [20].

Several papers have been published employing in vivo murine models demonstrating the contribution of purified VSELs to hematopoiesis, osteogenesis, angiogenesis, cardiomyocytes, and pulmonary alveolar epithelium [9, 11, 15, 21–25]. The presence of VSELs-derived chimerism in several organs after transplantation of these cells indicates their potential to differentiate across germ layers.

We demonstrated that quiescent VSELs enter the cell cycle in BM of male and female mice exposed to NAM and pituitary or gonadal sex hormones [26]. These data were intriguing as they provided evidence that adult murine BM contains a population of development early stem cells that express sex hormone receptors and that the molecular signature of these cells resembles the genomic state of migratory PGCs. In particular, quiescent VSELs proliferated in response to Follicle-Stimulating Hormone (FSH) + NAM stimulation [20, 27]. Therefore, to shed more light on this unique population of stem cells, we performed proteomic analysis of expression profiles in BM steady-state conditions VSELs (SSC-VSELs) isolated from control untreated mice and BM-VSELs purified from mice injected for 10 days with FSH + NAM (FSH + NAM VSELs).

We present data that VSELs express a unique panel of protein expression that only partially overlaps with the proteome of BM -derived hematopoietic stem cells (HSCs) and hematopoietic mononuclear cells (MNCs). The proteome of murine VSELs is characterized by the high number of proteins involved, e.g., in early development, cell division, transcription, signal transduction, cytoskeletal organization, and immune response. This data also further supports the developmental relationship of these cells to the germ lineage [17, 19]. Moreover, these investigations are in frame with United Nations Sustainable Development Goal 3 to ensure healthy lives and promote well - being for all at all ages.

## Materials and Methods

### Animals

6-week-old C57BL/6 male mice were divided into two groups for the study of proteomes at the steady-state conditions (SSC group) and after in vivo expansion of stem cells by repeated injections of Follicle-Stimulating Hormone (FSH) in combination with nicotinamide (NAM) (F + N group). FSH in dose 5 IU/mouse for and NAM 100 mg/kg were administrated intraperitoneal every 24 hours. Mice from the SSC group were injected in the same manner with PBS. After the 10th dose, mice were euthanized, and bone marrow was isolated for further preparation. The study was approved by the Local Ethical Committee for Experiments on Animals in Szczecin (2/2013 and 16/2014).

### In Vivo BrdU Treatment

Mice were intraperitoneally (i.p.) injected daily with 1 mg of BrdU solution, and the final injection of BrdU was 1 hour before euthanizing the animals. MNCs were subsequently isolated from BM after lysis of erythrocytes and immunostained for expression of Sca-1, CD45, and Lin markers, as reported previously [3], as well as for the presence of BrdU (FITC BrdU Flow Kit; BD Pharmingen, USA) [26]. Samples were analyzed with a Navios flow cytometer (Beckman Coulter, USA).

### Cell Isolation and Fluorescence-Activated Cell Sorting (FACS) of VSELs and HSCs

Bone marrow was isolated from tibias and femurs. Red blood cells were removed by lysis (RBC Lysis, BD Pharmingen), and MNCs were washed to remove a lysis buffer. Obtained cells stained with fluorescence-labeled antibodies against Lin markers (CD45R/B220, Gr-1, CD11b, TCR β, TCR γδ, Ter119), Sca-1, and CD45 (BD Pharmingen). The isolation of VSELs (Sca-1^+^Lin^−^CD45^−^) and HSCs (Sca-1^+^Lin^−^CD45^+^) was performed using multiparameter live-cell sorting (MoFlo Astrios, Beckton Dickinson, USA).

### Cell Lysates Preparation and Protein Digestion

5000 VSELs were isolated for each independent biological sample (n = 5). Cell pellets were lysed using RIPA buffer without protease/phosphatase inhibitors to avoid the formation of unspecific peaks on a chromatogram. Next the proteins subjected to heat denaturation at 98 °C for 1 h and ultrasound treatment on ice for 1 h. Proteins were precipitated with ice-cold acetonitrile LC-MS grade (Merck Millipore, USA), centrifuged at 18,000 x g for 30 min at −10 °C, and dried for 10 min at RT using Eppendorf Concentrator plus (Eppendorf, Germany). Then proteins were re-dissolved in 40 mM ammonium bicarbonate solution, reduced with 20 mM DTT solution, and alkylated with 40 mM iodoacetamide solution (Sigma Aldrich, USA). In-solution trypsin digestion was performed overnight at 37 °C with shacking, and the digestion was stopped by adding 0.1% formic acid.

### LC-MS Proteome Analysis

LC-MS analysis was carried out with the use of nanoUHPLC (nanoElute, Bruker) coupled with CaptiveSpray (Bruker) to ESI-Q-TOF mass spectrometer (Compact, Bruker). Two-Column separation method was used, i.e., pre-column (300 μm × 5 mm, C18 PepMap 100, 5 μm, 100 Å, Thermo Scientific) and Bruker FIFTEEN separation column with CSI fitting (75 μm × 150 mm, C18 1.9 μm) in gradient 2% B to 35% B in 90 minutes with the 300 nL/min flow rate. Following mobile phases were used: A – 0.1% formic acid in water; B – 0.1% formic acid in ACN. Ionization of the samples was carried out at a gas flow of 3.0 L/min, the temperature of 150 °C, and the voltage of the capillary 1600 V. The quadrupole energy was set to 5.0 eV and collision chamber energy to 7.0 eV with an ion transfer time of 90 μs. The ions were analyzed in the positive polarity mode in the range 150-2200 m/z, with the acquisition frequency of the 4 Hz spectrum and the autoMS/MS system. The collected spectra were analyzed and calibrated (Na Formate) in DataAnalysis software (Bruker) and then, after extracting the peak list, identified in ProteinScape (Bruker) using the MASCOT server. Proteins were identified using the online SwissProt and NCBI_prot databases, and their annotation and biological significance were determined using Reactome.org and String.org.

### Statistical Analysis

At least five independent replicates were performed for all experiments, and data were analyzed using GraphPad Prism 7 software (GraphPad, USA). The significance of differences between experimental groups was analyzed using Student’s t test, and a P value below 0.05 was considered significant.

## Results

### In Vivo Expansion of Murine BM VSELs

**Fig. 1:**
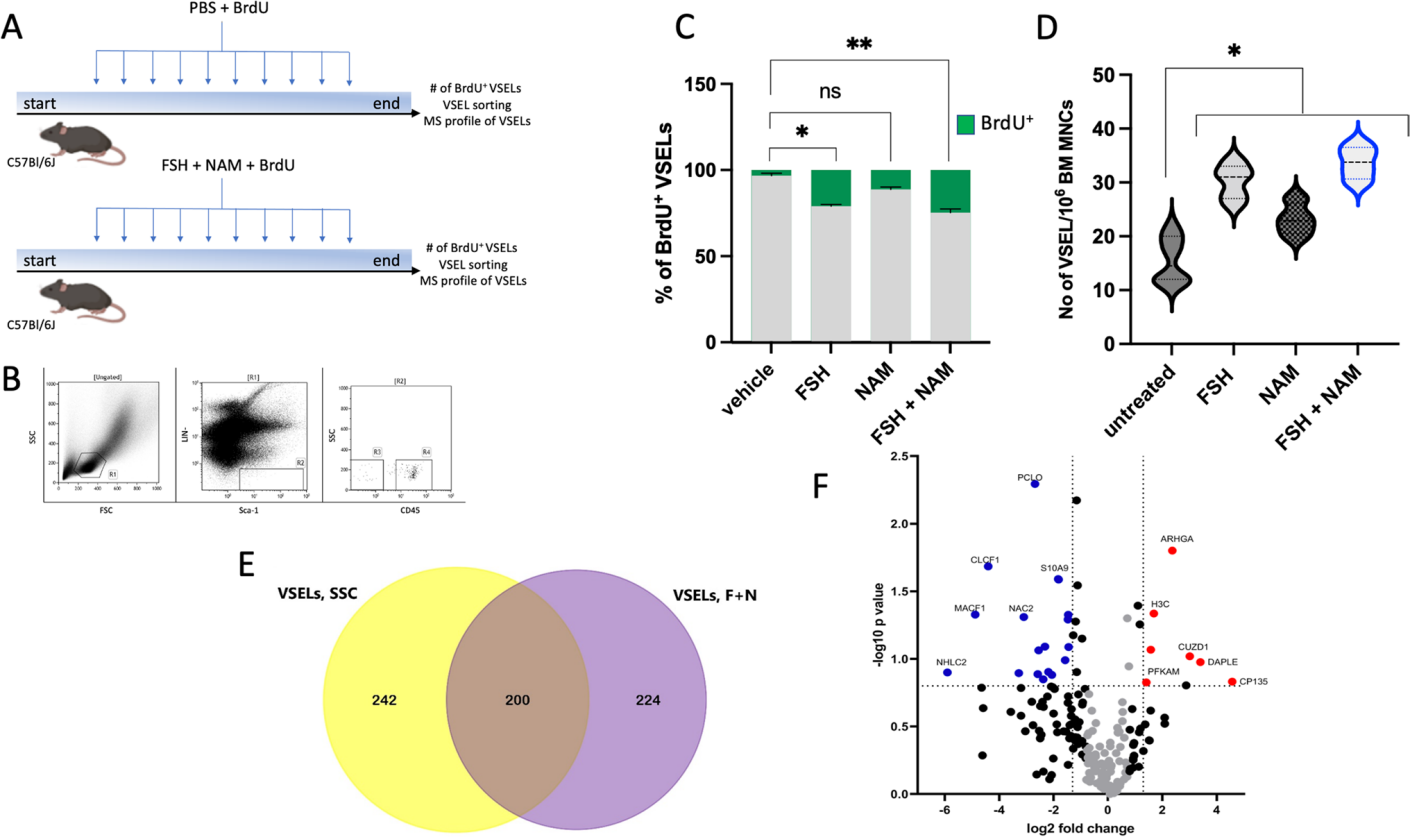
**A** Schematic presentation of experimental groups**. B** Representative example of cytometric analysis used to evaluate the number of VSELs in the murine bone marrow. The R1 gate contains small, agranular cells. Gate R2 contains Sca-1+ / Lin − cells which are visualized on the next plot as CD45 negative (VSEL) and CD45 positive (HSC) cells. **C** BrdU incorporation into mBM VSEL DNA in response to administration of FSH and NAM in vivo*.* The percentage of cells that showed proliferative activity and incorporated BrdU is shown in *green, and* cells that did not proliferate (BrdU-negative) are shown in *light grey*. The results obtained after stimulation with FSH and NAM were compared to those obtained from the control. *p < 0.05; control group n = 14, study groups n = 8. **D** Changes in the total number of BM VSELs (Sca-1+/CD45-/Lin-) per 10^6^ MNC after administration of FSH and NAM. Quantitative analysis of cells was done using Kaluza software (Beckman Coulter, USA). *p ≤ 0.05, **p ≤ 0.005 compared with control. **E** Venn diagram of VSELs proteomics analysis. Comparison of the proteins annotated at the steady-state conditions and in expanded VSELs (F + N). **F** Volcano plot displaying the distribution of all common proteins in VSELs datasets SSC vs. F + N (n = 200) with relative protein abundance (log2 fold change) plotted against its significance level (negative log10 P value), showing significantly (P < 0.05) increased (red dots) and decreased (blue dots) proteins in studied VSELs datasets

Mice employed in our studies injected with vehicle combined with BrdU or with FSH + NAM combined with BrdU tolerated well daily injections (Fig. 1A**).** VSELs were analyzed and sorted from murine BM by employing multiparameter staining as the population of very small Sca-1^+^lin^−^CD45^−^ cells, as shown in Fig. 1B in R3. The region R4 shows Sca-1^+^lin^−^CD45^+^ cells enriched in HSCs. We noticed that quiescent VSELs entered the cell cycle, and 20-25% of these cells accumulated BrdU (Fig. 1C**)**. At the same time, we noticed that these cells expanded in vivo in murine BM as the number of these cells increased >3 times as compared to control untreated animals (Fig. 1D).

### Quantitative Proteomic Identification of Proteins in Murine BM-Derived VSELs

**Supplementary Fig.** **1** shows Venn diagrams of proteomes from BM-derived SSC-VSELs, HSCs, and MNCs at the steady-state conditions (left panel) and FSH + NAM VSELs (right panel). As shown, VSELs present a unique panel of protein expression that only partially overlapped with the proteome of HSCs and MNCs. Furthermore, FSH + NAM VSELs isolated from animals receiving a repeated injection of FSH and NAM display a unique proteomic profile. The number of annotated common proteins to VSELs, HSCs, and MNCs was 69 at the steady-state conditions and 61 after FSH + NAM treatment (**Supplementary Fig.** **1**). These common proteins mainly involve cell cellular transport, immunity, apoptosis, and transcription.

### Comparative Analysis of Proteins Identified in VSELs Datasets at the Steady-State Conditions and after FSH + NAM Stimulation

**Fig. 2:**
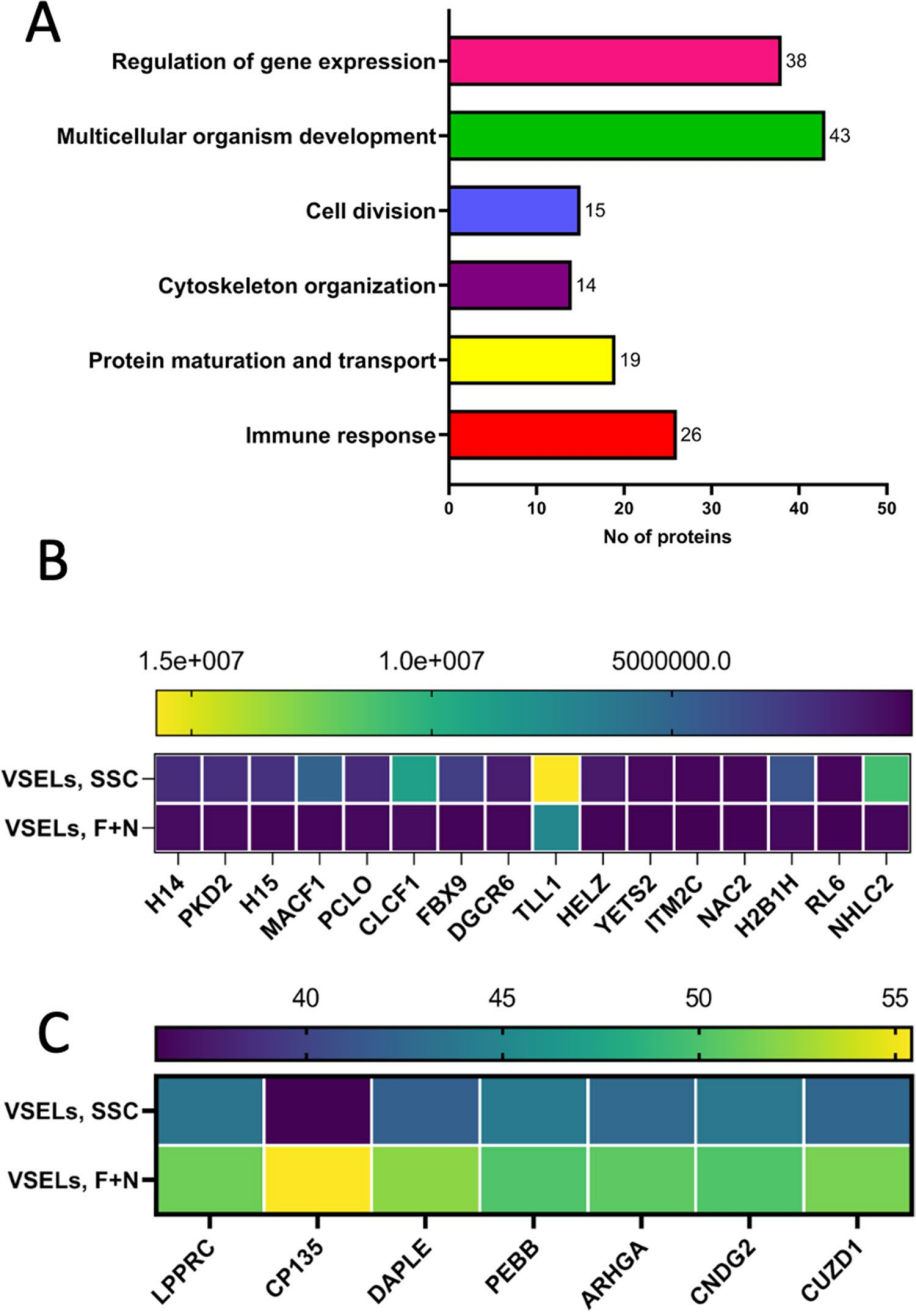
Analysis of annotated proteins common to VSELs in the steady-state conditions and after the F + N challenge. **A** Functional classification of gene ontology (GO) of proteomic data in VSELs SSC and VSELs F + N datasets. Comparative analysis of annotated common proteins corresponding to GO biological processes regulating immune response, proteins maturation and transport, cytoskeleton organization, cell division, and regulation of gene expression in both datasets. **B** The mean normalized heat map displaying the abundance of annotated down-regulated proteins in label-free proteomics analysis of VSELs at SSC and F + N treatment. **C** The mean normalized heat map presents statistically significant up-regulation of proteins in expanded VSELs

We noticed that up to 200 annotated proteins were common for both experimental groups SSC-VSELs and FSH + NAM VSELs (Fig. 1E). We found that 20 proteins were down-regulated and 7 proteins up-regulated in a statistically significant manner in FSH + NAM VSELs compared to SSC-VSELs (Fig. 1F). The most enriched GO biological pathways and number of proteins involved in regulating gene transcription, multicellular organism development, cell cycle and division, cytoskeleton organization, protein maturation, transport, and immunity are presented in Fig. 2A.

Figure 2B shows selected down-regulated and up-regulated proteins in FSH + NAM VSELs. We observed substantial down-regulation of NHL repeat-containing protein 2 (NHLC2) (60 fold), microtubule-actin cross-linking factor 1 (MACF1) (29 fold), and F-box only protein 9 (FBX9) (24 fold). Moreover, we observed downregulation of histone 1.4 (H14), histone 1.5 (H15), and histone H2B type 1 (H2B1H) was downregulated in FSH + NAM VSELs. Also, we have detected downregulation of several proteins involved in histone acetylation/deacetylation and histone/DNA methylation by analyzing pathways in FSH + NAM VSELs compared to SSC-VSELs. Specifically, YEATS domain-containing protein 2 (YETS2) was down-regulated 9 folds (Fig. 2B).

In contrast, as mentioned above 7 annotated common proteins were significantly up-regulated in FSH + NAM VSELs compared to SSC-VSELs (Fig. 2C). This includes proteins involved in early development, such as CUB and zona pellucida-like domain-containing protein 1 (CUZD1), Protein Daple (DAPLE), core-binding factor subunit beta (Cbfβ), and condensin-2 complex subunit G2 (CNDG2). The most up-regulated protein in the NAM + FSH VSELs dataset was the centrosomal protein of 135 kDa (CP135) required for centriole biogenesis. Moreover, we also found that Rho guanine nucleotide exchange factor 10 (ARGHA) required for cytoskeleton organization and cytokinesis was up-regulated in expanded VSELs.

### Functional Classification and Annotation of Proteins Regulating VSELs Cell Cycle and Transcription

**Fig. 3:**
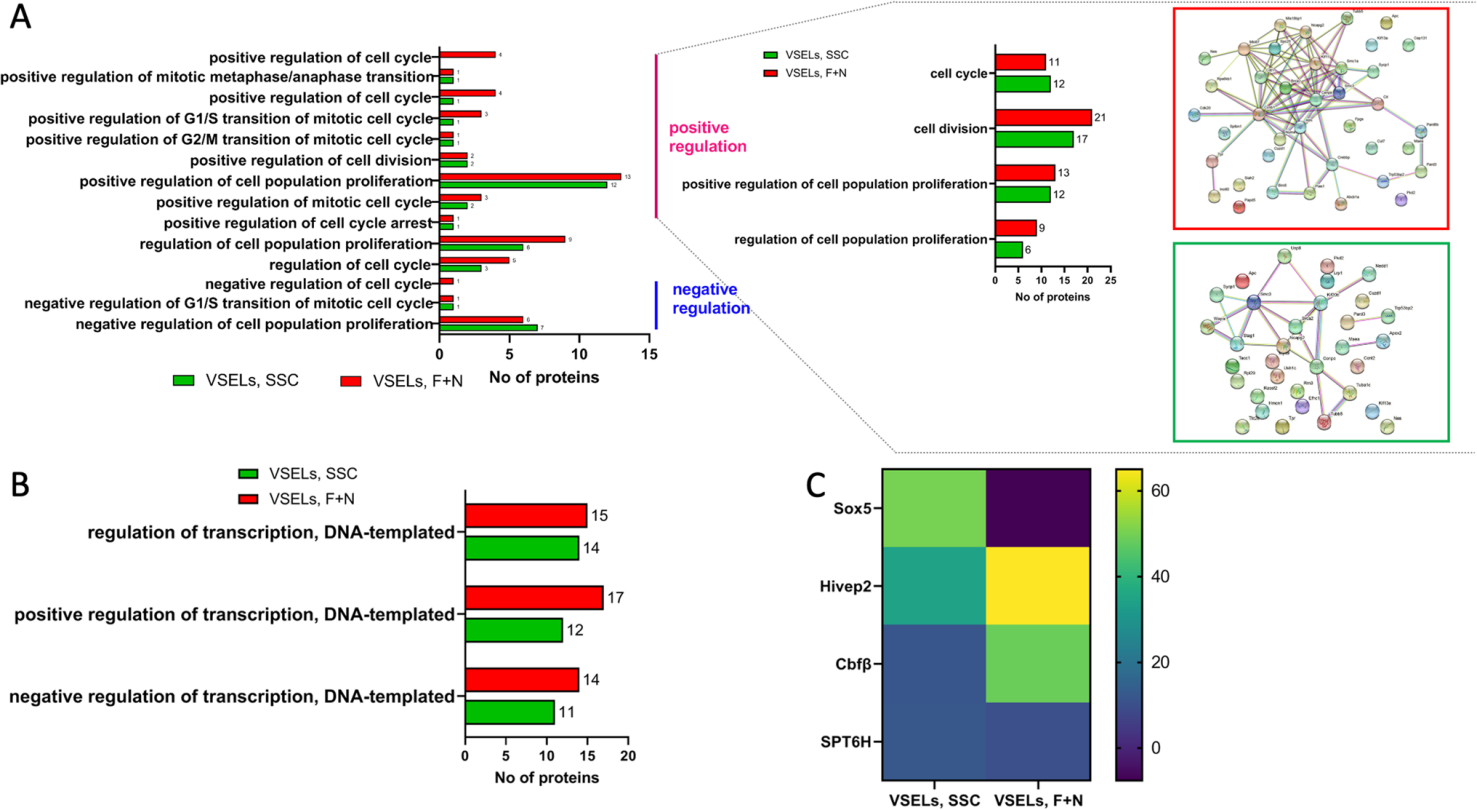
Functional enrichment analysis of annotated proteins involved in the regulation of cell cycle and transcription in VSELs. **A** GO functional annotation histogram of the cell cycle and cell proliferation proteins. At the right, the STRING (version 11.5) interaction networks demonstrate cell cycle proteins annotated in VSELs F + N dataset (upper map) and in VSELs SSC (lower map). Proteins are shown as nodes, and each edge represents an interaction. **B** GO enrichment analysis of transcriptional proteins in both VSELs datasets. **C** The mean normalized heat map presents a statistically significant expression of transcriptional factors in VSELs at SSC and in VSELs treated with F + N

By performing GO enrichment analysis, we noticed an increase in the number of proteins involved in cell division, the transition between cell cycle checkpoints, and cell cycle arrest in FSH + NAM VSELs (Fig. 3A). STRING maps visualization of cell cycle proteins present 42 proteins in FSH + NAM VSELs in the red rectangle and 31 proteins in the green rectangle in SSC-VSELs. The number of proteins positively regulating cell cycle and G1/S transition of the mitotic cell cycle was higher in FSH + NAM VSELs compared to SSC-VSELs, including G2/mitotic-specific cyclin-B1 (CCNB1) that regulates G2/M transition), forkhead box protein G1 (FOXG1), ankyrin repeat domain-containing protein 17 (ANR17) and fibroblast growth factor receptor 2 (FGFR2).

About 15% of all annotated proteins in both VSELs datasets are identified as transcriptional proteins, and FSH + NAM VSELs are characterized by the increased number of transcriptional proteins correlated with cell cycle progression (data not shown). We also noticed (Fig. 3B) a significant increase in the number of proteins involved in positive regulation of DNA-templated transcription: accordingly, 17 proteins were identified in FSH + NAM VSELs and 12 in SSC-VSELs. At the same time, 14 proteins involved in the negative regulation of DNA-templated transcription were upregulated in FSH + NAM VSELs as compared to 11 genes in SSC-VSELs.

Simultaneously, we detected an increased number of transcription factors in the FSH + NAM VSELs dataset. By employing the Uniprot database, we identified several early developmental transcription factors, including POU domain class 2 transcription factor 2 (PO2F), transcription factor SOX-9, hypoxia-inducible factor 3-alpha, transcription elongation factor SPT6, transcription initiation factor TFIID subunit 3 (TAF3), transcription factor HIVEP2, eukaryotic translation initiation factor 3 subunit I (EIF3I), transcription initiation factor TFIID subunit 11 (TAF11), and transcription factor SOX-5. Interestingly as it is shown in Fig. 3C, Sox5 expression was downregulated, and expression of HIVEP2 and Cbfβ were increased in FSH + NAM VSELs compared to SSC-VSELs (Fig. 3C).

### Functional Classification and Annotation of Proteins Involved in VSEL Signal Transduction

**Fig. 4:**
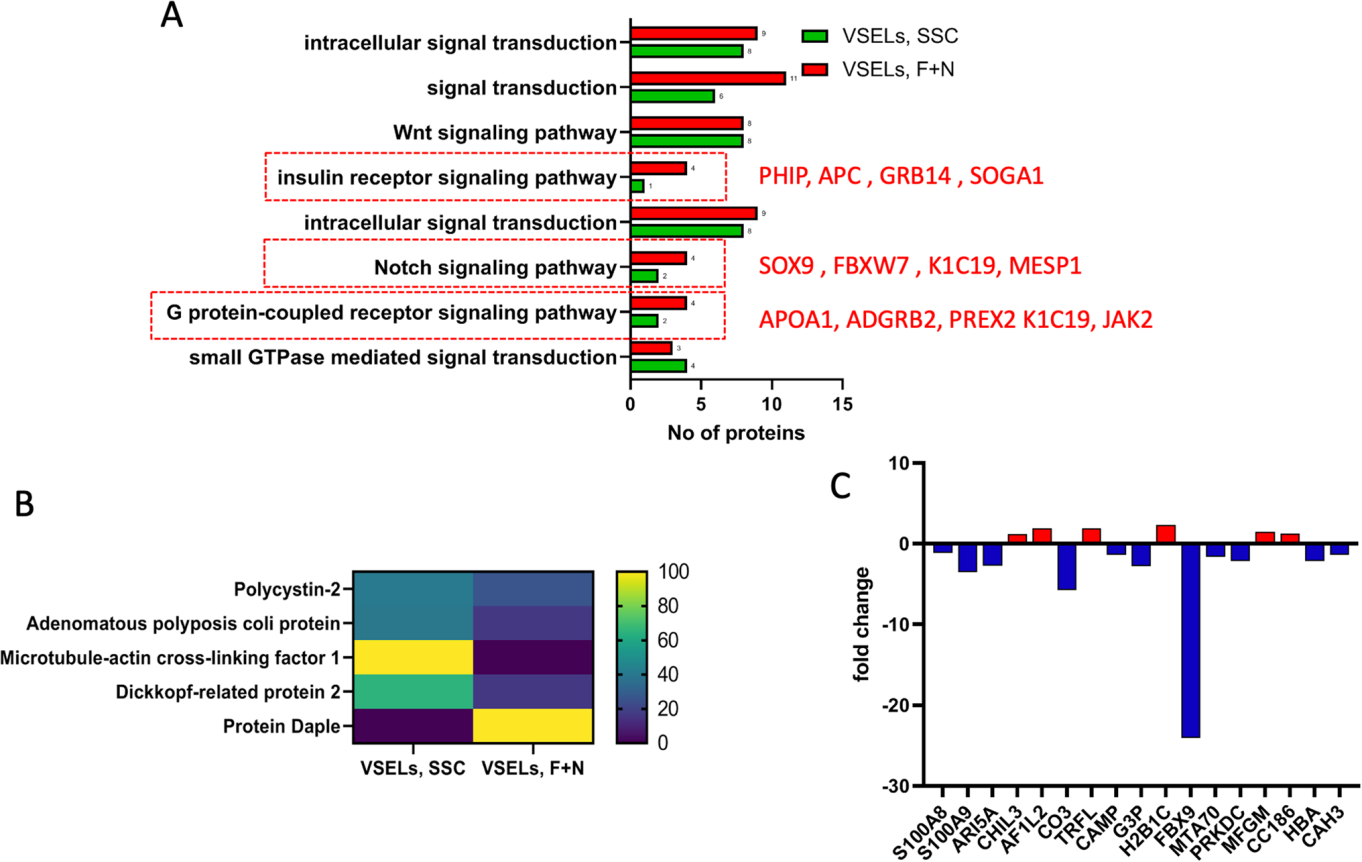
Proteomic analysis reveals alterations in cell signaling and immunity-associated proteins in VSELs SSC vs. VSELs F + N. **A** GO enrichment results of most abundant signaling pathways in both VSELs datasets. **B** The mean normalized heat map displays the abundance of annotated proteins playing a pivotal role in the Wnt signaling pathway. **C** The fold changes in expression of annotated immune proteins were identified in both VSELs datasets

In the Fig. 4A**,** there are shown proteins detected in VSELs datasets involved in various signaling pathways. We have identified 72 proteins in the FSH + NAM VSELs data set and 60 proteins in SSC-VSELs. The most significant proteins belong to Wnt, Notch, insulin receptor, and G protein coupled signaling pathways.

The total number of proteins involved in the Wnt signaling or regulating this pathway was comparable in both VSELs datasets. However, except for protein Daple the common proteins involved in the Wnt signaling pathway are downregulated in the FSH + NAM VSELs dataset as compared to SSC-VSELs (Fig. 4B). Accordingly, Daple was 10 folds up-regulated in expanded FSH + NAM VSELs.

In contrast intensity of adenomatous polyposis coli protein and dickkopf-related protein 2 peptides decreased 2.2 and 1.6-fold, respectively, in FSH + NAM VSELs as compared to SSC-VSELs. In addition, polycystin-2 expression was reduced 7-fold in the FSH + NAM VSELs. Of note, the expression of microtubule-actin cross-linking factor-1 is decreased 29 times in FSH + NAM VSELs as compared to SSC-VSELs with a statistical significance of p < 0.049. Interestingly the expression of the negative regulator of canonical Wnt signaling Tle2 (transducin-like enhancer protein 2) was identified only in SSC-VSELs dataset.

While expression and upregulation of genes involved in cell development and proliferation were to be expected, we were somehow surprised by the expression of proteins related to innate immunity (Fig. 2A). Accordingly, we detected complement component C3, small calcium-binding proteins S100-A8 and S100-A9, antimicrobial peptide cathelicidin, and AT-rich interactive domain-containing protein 5A. However, not surprisingly the most identified common immune proteins were slightly down-regulated in expanded FSH + NAM VSELs (Fig. 4C).

### Functional Enrichment Analysis of Developmental Proteins Identified in BM VSELs

**Fig. 5:**
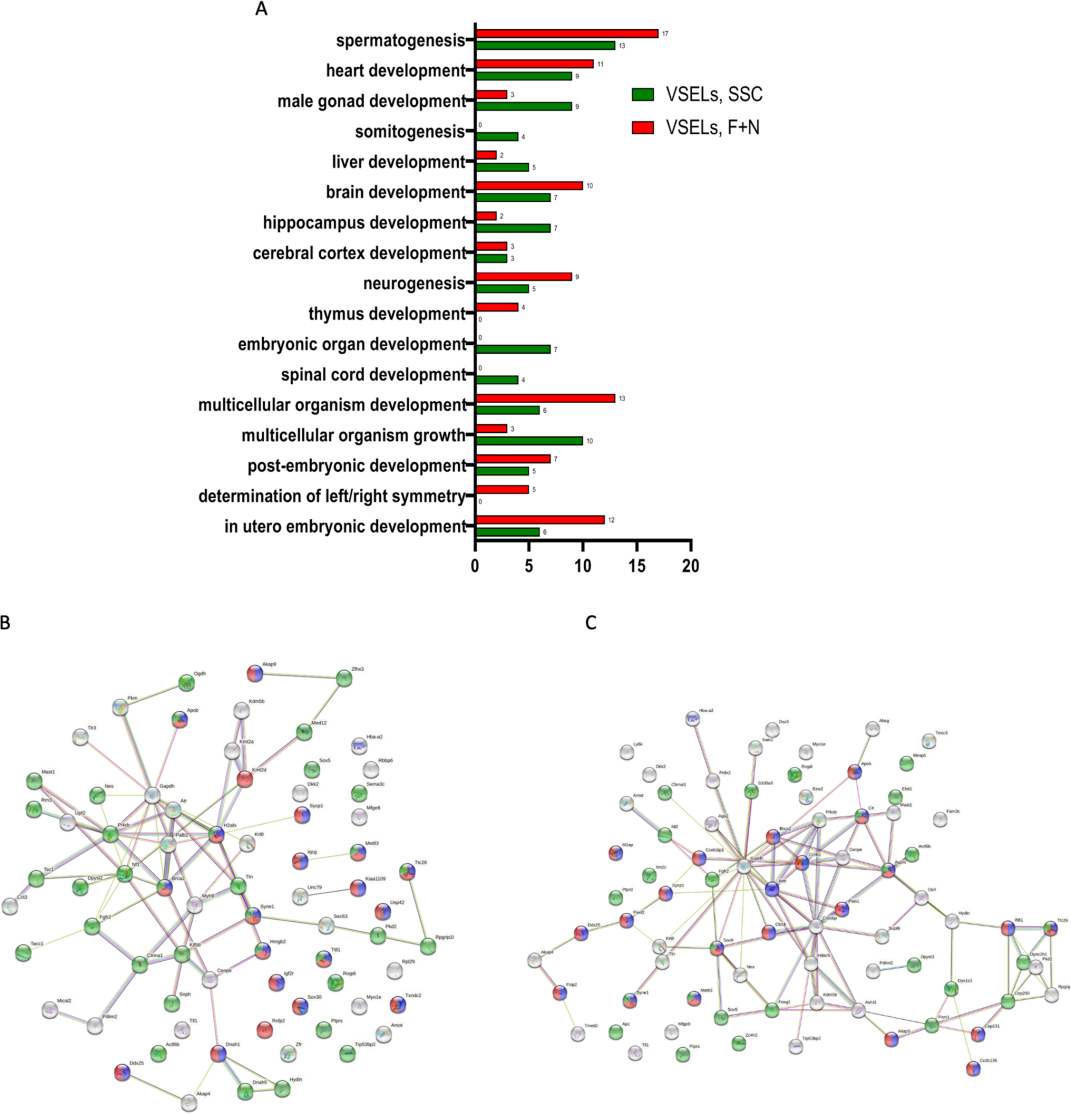
Proteomic analysis of differentially expressed developmental proteins in VSELs SSC and VSELs F + N. **A** GO enrichment analysis of proteins responsive for cell/organ/organism development in expanded VSELs and at the steady-state conditions. **B** The STRING (version 11.5) interaction network of developmental proteins in VSELs SSC. Proteins are shown as nodes, and each edge represents an interaction. The proteins classified to GO biological process GO:0007283 are shown for Spermatogenesis colored blue, GO:0007276 Gamete generation colored red, and GO:0007399 Nervous system development colored green. **C** The STRING (version 11.5) interaction network of developmental proteins in VSELs F + N. Proteins are shown as nodes, and each edge represents an interaction. The proteins classified to GO biological process GO:0007283 are shown for Spermatogenesis colored blue, GO:0007276 Gamete generation colored red, and GO:0007399 Nervous system development colored green

VSELs are primitive stem cells; thus, as anticipated, almost 20% of the whole proteome was comprised of developmental proteins. We postulated developmental relationship of these cells to migrating PGCs, and herein using the functional enrichment approach, we found that most of the annotated proteins in studied by us male VSELs were involved in spermatogenesis in both VSELs datasets (Fig. 5A). Nevertheless, the number of spermatogenic proteins seems to be higher in FSH + NAM VSELs than SSC-VSELs, what confirms that these cells are highly responsive to stimulation by FSH. Accordingly, 17 proteins were identified in FSH + NAM VSELs dataset vs. 13 proteins in SSC- VSELs dataset.

Since we performed experiments with male mice, it is not surprising that we found several proteins involved in spermatogenesis and male gonad development. Accordingly, we found proteins responsible for germ-line stem cell population maintenance (e.g., piwi-like protein 2 in FSH + NAM VSELs), single fertilization (e.g., lysine-specific demethylase 5B, and protein-lysine acetyltransferase CREBBP in FSH + NAM VSELs) and egg-sperm recognition protein (e.g., CUB and zona pellucida-like domain-containing protein 1). Moreover, under the steady-state condition, we were able to detect the presence of Sox 30, DDX25, AKAP9, BRCA2, and MPRI. Sox 30 is required for activation of the post-meiotic gene expression involved in spermatogenesis, whereas DDX25, AKAP9, BRCA2, and MPRI are critical for spermatid development, spermatogenesis, and Sertoli cell maturation in mice, and oocyte maturation. We also detected the presence of KDM5B (Lysine-specific demethylase 5B), the first known histone demethylase affecting female fertility (Fig. 5B). Furthermore, we detected in the FSH + NAM VSELs dataset higher expression of nesprin-1 (SYNE1), which might be involved in nuclear remodeling during the formation of the sperm head, SYCP1 (synaptonemal complex protein 1), which is a major component of the transverse filaments of synaptonemal complexes, formed between homologous chromosomes during meiotic prophase. Of note, A-kinase anchor protein 9 (AKAP9) was also detected in FSH + NAM VSELs, which is essential for spermatogenesis and Sertoli cell maturation in mice. Interestingly, the most significant changes in the number of annotated developmental proteins of the spermatogenesis network were identified in FSH + NAM VSELs (Fig. 5C).

We also identified proteins involved in neurogenesis and neural structure development (Fig. 5B, C) and 29 proteins were identified in SSC-VSELs and 22 in FSH + NAM VSELs. It is not surprising as neurogenesis is one of the earliest genetic programs activated in embryonic development. Nevertheless, the intensities of common neurogenic proteins were decreased in the expanded VSELs as compared to steady-state VSELs. Accordingly, actin-like protein 6B, nestin, protein-tyrosine phosphatase delta, and adenomatous polyposis protein (APC) were downregulated 5,2; 1.7; 2.5, and 2.2 times respectively. Moreover, we detected more proteins involved in nervous system development in SSC-VSELs compared with the FSH + NAM VSELs. As was mentioned above, actin-like protein 6B and nestin were annotated in both datasets. These proteins play a pivotal role in neural progenitor cell self-renewal. In the SSC-VSELs dataset were also annotated, e.g., DOCK7 (dedicator of cytokinesis 7), netrin-G2, ectoderm-neural cortex protein 1 (ENC-1), and neurofibromin (Nf1) responsible for developing neural crest-derived structures, Ten-3. On the other hand, FSH + NAM VSELs are characterized by proteins promoting neuron differentiation (zinc finger C4H2 domain-containing protein, R-PTP-delta, basic leucine zipper, and W2 domain-containing protein 2), migration (dynein axonemal assembly factor 4, Unc-33-like phosphoprotein 1) and the development of the central nervous system (citron Rho-interacting kinase).

Furthermore, both FSH + NAM VSELs and SSC-VSELs express proteins involved in embryogenesis and post-embryonic development (Fig. 5A). VSELs express also genes involved in heart developments. We also identified the hemoglobin subunit epsilon (HBE1) in both VSELs datasets but not in other BM-derived cells. This type of hemoglobin is known only to be expressed at the early stages of mammalian embryonic development. This supports hematopoietic differentiation of these small cells. Moreover, we noticed expression of several genes involved in thymus and immune system development.

## Discussion

The seminal message of this work is that VSELs express a unique panel of protein expression that only partially overlapped with the proteome of BM-derived HSCs and hematopoietic MNCs, and that they respond to NAM + FSH stimulation by increasing the expression of factors involved, e.g., in developmental organogenesis, signal transduction, insulin signaling, cytoskeleton organization, cell adhesion, inhibiting apoptosis, promoting protein transport and stabilization, protein phosphorylation and ubiquitination, involved in DNA repair, immune response, and regulation of circadian rhythm. Furthermore, our current data further supports germ-lineage origin of these cells [20, 28].

It is reported that VSELs are a quiescent population of early development stem cells deposited in adult tissues, including BM, and reside in the dormant state due to changes in the expression of paternally imprinted genes [16, 18, 29]. Recently we developed ex vivo expansion of these cells in the culture system, with NAM + FSH being the most critical components of the expansion medium [20]. This system was based on our data that demonstrated a link between VSELs and migrating PGCs [17] and a crucial role of NAM as a DNA modifying agent that promotes the re-establishment of proper somatic imprinting at erased DMRs of parentally imprinted genes [20, 27, 30]. Based on this, we learned that we could force quiescent murine BM VSELs to enter the cell cycle and proliferate in the presence of NAM + sex hormones, including FSH. The number of VSELs that entered in vivo in mice the cell cycle after repeated injection of FSH was ~25-30%. Moreover, what is interesting female patients treated because of infertility with FSH mobilized VSELs into peripheral blood [31], which provides additional evidence that FSH affects the biology of these quiescent cells [27].

In the current report, we enumerated the number of VSELs in murine BM after exposure to FSH + NAM injections and found that after 10 days of repeated daily injection, the number of VSELs increased in BM > 3 times. Both SSC-VSELs, and FSH + NAM VSELs were subsequently purified from murine BM by FACS. As mentioned above, we found that VSELs express a unique panel of proteins that only partially overlapped with the proteome of HSCs and MNCs. Using the Gene Ontology resources and GO-CAM model of analysis, which is a combination of standard GO annotations allowing to produce a network of annotations, we were able to identify several GO biological processes, SSC-VSELs and FSH + NAM VSELs, including multicellular organism development, organogenesis, regulation of gene expression, signal transduction, Wnt signaling pathway, cytoskeleton organization, cell adhesion, negative regulation of the apoptotic process, response to extra- and intracellular stimuli, protein transport and stabilization, protein phosphorylation and ubiquitination, DNA repair, immune response, and regulation of circadian rhythm.

Moreover, while analyzing the signal transduction pathways, we find that the largest group of proteins expressed in VSELs belongs to the Wnt signaling pathway, which is one of the most crucial developmental signaling pathway that controls cell fate decisions and tissue patterning during early embryonic development. However, we observed downregulation of Wnt signaling pathway proteins FSH + NAM VSELs compared to SSC-VSELs, what could be explained by the proceeding differentiation of thyese cells after in vivo exposure to FSH + NAM. Nevertheless, at the same time, protein Daple that interacts with Dishevelled and activates Rac1 mediated non-canonical Wnt signaling pathway required for cell migration [32] was upregulated in FSH + NAM VSELs. This observation supports several observations that these cells upon activation, may migrate to damaged tissues and regenerate them. At the same time, we also noticed a slight decrease in FSH + NAM VSELs expression of adenomatous polyposis coli protein (APC) and dickkopf-related protein 2 (DKK2), that act as repressors of the Wnt signaling pathway. [33].

Among proteins significantly downregulated in expanded VSELs is microtubule-actin cross-linking factor-1 involved in cytoskeleton network dynamics. In addition, we also detected expression of leucine-rich PPR motif-containing protein, CUB and zona pellucida-like domain-containing protein 1, condensin-2 complex subunit G2, and core-binding factor subunit beta (Cbfβ). What is important, the last two proteins are involved in stem cell maintenance and early stages of differentiation [34, 35]. The regulatory subunit of the condensin-2 complex establishes mitotic chromosome architecture and is involved in the physical rigidity of the chromatid axis [36]. It is required for early embryonic development and is essential for viability and expansion of the implanting blastocyst to the uterus. We also detected in FSH + NAM VSELs, 17 proteins involved in the positive regulation of DNA transcription. By utilizing the Reactome pathways, we identified several early developmental transcription factors including, POU domain, class 2, transcription factor 2 (PO2F), transcription factor SOX-9, hypoxia-inducible factor 3-alpha, transcription elongation factor SPT6, transcription initiation factor TFIID subunit 3 (TAF3), transcription factor HIVEP2, eukaryotic translation initiation factor 3 subunit I (EIF3I), transcription initiation factor TFIID subunit 11 (TAF11), and transcription factor SOX-5.

As expected, we detected in SSC-VSELs presence of many genes involved in spermatogenesis, including Sox 30, DDX25, AKAP9, BRCA2, and MPRI. Of note, while Sox 30 is required for activation of the post-meiotic genes involved in spermiogenesis, DDX25, AKAP9, BRCA2, and MPRI proteins are critical for spermatid development and maturation of Sertoli cells and oocytes. We also detected the presence of Lysine-specific demethylase 5B (KDM5B), the known histone demethylase affecting female fertility. It has been reported that KDM5B^−/−^ female mice have a significantly reduced fertility rate [37]. Moreover, NAM + FSH VSELs expressed nesprin-1 (SYNE1), involved in nuclear remodeling during sperm head formation during spermatogenesis [38]. Moreover, SYNE1 serves as a multi-isomeric modular protein that forms a linking network between organelles and the actin cytoskeleton to maintain the subcellular spatial organization. NAM + FSH VSELs also display higher expression of synaptonemal complex protein 1 (SYCP1), a significant component of the synaptonemal complexes formed between the transverse filaments homologous chromosomes during meiotic prophase during gametogenesis. Moreover, SYCP1 is required for normal meiotic chromosome synapsis during the mouse’s oocyte and spermatocyte development and normal male and female fertility [39]. Of note, we also detected in NAM + FSH VSELs the presence of A-kinase anchor protein 9 (AKAP9) [40], essential for spermatogenesis and Sertoli cell maturation in mice - critical for the centrosomes association with the poles of the bipolar mitotic spindle during metaphase [41].

This data is intriguing for two reasons. Firstly, they confirm that SSC-VSELs express proteins involved in gametogenesis. Thus, our data confirm that BM could be a source of cells with potential differentiation into gametes [42–45]. Since our data were performed in male mice, it would be interesting to execute a similar proteomic analysis in female mice exposed to NAM + FSH. Moreover, population of small cells corresponding to VSELs have been identified ion murine and human gonads. I few elegant papers it has been postulated that these cells could be precursors of gametes [5, 13, 31, 46].

Murine VSELs are also characterized by the expression of proteins belonging to nervous system development. Accordingly, 29 proteins were annotated at the steady-state conditions and 22 in expanded VSELs. Of note, it is not surprising that we detected more proteins involved in nervous system development in SSC-VSELs as compared to FSH + NAM-VSELs, including the expression of nestin and actin-like protein 6B, which plays a pivotal role in neural progenitor cells’ self-renewal [47]. In the SSC-VSELs dataset, we also annotated DOCK7, an important protein for adult neurons and neural progenitor cell proliferation [48]; netrin-G2 is involved in controlling patterning and neuronal circuit formation [49]; ectoderm-neural cortex protein 1 (ENC-1) and neurofibromin (Nf1) are responsible for the development of neural crest-derived structures [50]; teneurin 3 (Ten-3) involved in neural development. On the other hand, NAM + FSH VSELs were characterized by proteins promoting neuron differentiation (zinc finger C4H2 domain-containing protein, R-PTP-delta, basic leucine zipper, and W2 domain-containing protein 2), migration (dynein axonemal assembly factor 4, Unc-33-like phosphoprotein 1) and the development of the central nervous system (citron Rho-interacting kinase). Nevertheless, the intensities of common neurogenic proteins decreased in the NAM + FSH-VSELs dataset compared to SSC-VSELs. This, again, may be explained by their differentiation after stimulation by NAM + FSH. To support this, actin-like protein 6B, nestin, protein-tyrosine phosphatase delta, and APC were downregulated 5,2; 1.7; 2.5 and 2.2 times, respectively in FSH + NAM VSELs. Nevertheless, the expression of neuronal markers in VSELs supports their potential involvement in neurogenesis [51].

While expression and upregulation of proteins involved in cell proliferation were to be expected, we were somehow intrigued by the expression of proteins related to innate immunity. Accordingly, we detected complement component C3, small calcium-binding proteins S100-A8 and S100-A9, antimicrobial peptide cathelicidin, and AT-rich interactive domain-containing protein 5A. The expression of the C3 component of the complement cascade in VSELs could suggest the potential involvement of intracellular complosome in regulating the biology of these cells [52–54]. We also noticed the expression of RNA-binding interactive domain-containing protein, which controls the inflammatory response by stabilizing selective inflammation-related mRNAs, such as IL6, STAT3, and TBX21, and to stem-loop structures located in the 3’UTRs of IL6, STAT3, and TBX21 mRNAs; that at least for STAT3 prevents binding of ZC3H12A to the mRNA stem-loop structure, thus inhibiting its degradation activity. The expression of innate immunity genes in BM-derived VSELs may support their immune-modulatory role in inhibiting GvHD in mice.

Finally, as we reported in the past murine and human VSELs give rise to functional HSCs. This explains why VSELs in our current study express at protein level beta-type chain of early mammalian embryonic hemoglobin known as hemoglobin subunit epsilon (HBE1), characteristic of primitive hematopoiesis [55]. Moreover, VSELs express also the Core Binding Factor β (CBFβ) subunit required for hematopoietic stem cell emergence, bone formation, and normal differentiation of lymphoid and myeloid lineage cells [56]. To support this notion, Cbfβ in complex with the RUNX family of transcription factors in the cell nucleus supports activation or.

repression of genes related to bone (RUNX2), hematopoiesis (RUNX1) and gastrointestinal (RUNX3) development [57].

In conclusion, VSELs express a unique panel of proteins that only partially overlapped with the proteome of the BM-derived HSCs and hematopoietic MNCs. They proliferate and differentiate in response to NAM + FSH stimulation and express genes involved in organogenesis and development, signal transduction, Wnt and insulin signaling, cytoskeleton organization, cell adhesion, inhibiting apoptosis, protein transport and stabilization, protein phosphorylation and ubiquitination, DNA repair, immune response, and regulation of circadian rhythm. This data further demonstrates the close relationship of these cells to germ lineage and the unique expression of proteins directing the development of all three germ layers. Based on this VSELs are potential candidates of early development stem cells that could be applied in regenerative medicine.

## Supplementary Information

High Resolution Image (TIFF 551 kb)

High Resolution Image (TIFF 397 kb)

## Data Availability

The authors confirm that the data supporting the findings of this study are available within the article and its supplementary materials. Upon request authors may provide *.mgf files with raw mass spectrometry data.
