# Supplementary figures and images for: Proteomic Analysis of Murine Bone Marrow Very Small Embryonic-like Stem Cells at Steady-State Conditions and after In Vivo Stimulation by Nicotinamide and Follicle-Stimulating Factor Reflects their Germ-Lineage Origin and Multi Germ Layer Differentiation Potential
Source: Stem Cell Rev Rep. 2022 Aug 20;19(1):120–32. doi: 10.1007/s12015-022-10445-6 (PMC9823037; doi:10.1007/s12015-022-10445-6)

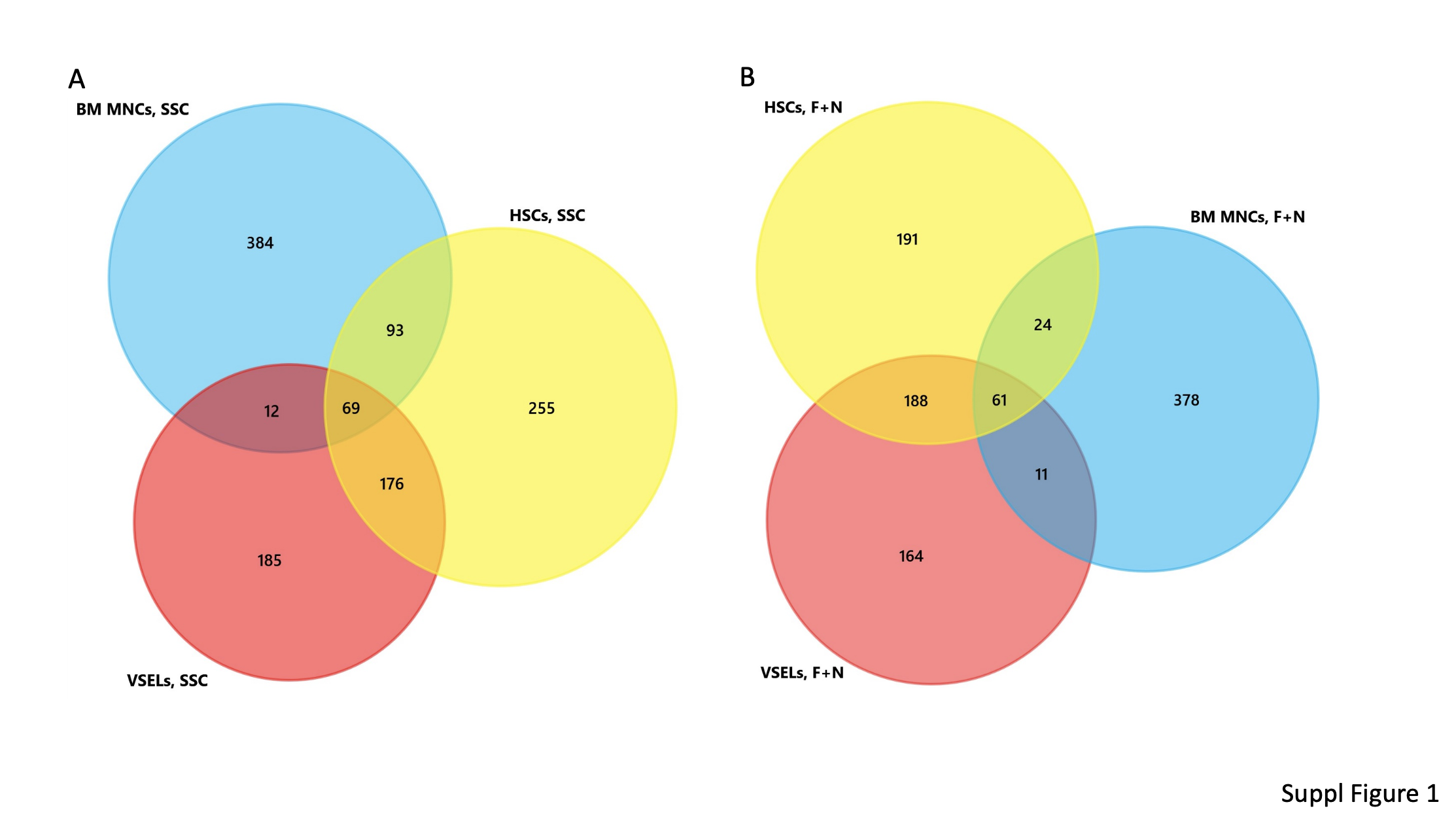

Supplement: Supplementary file 1 — High Resolution Image (TIFF 551 kb) [file 12015_2022_10445_MOESM1_ESM.tiff]

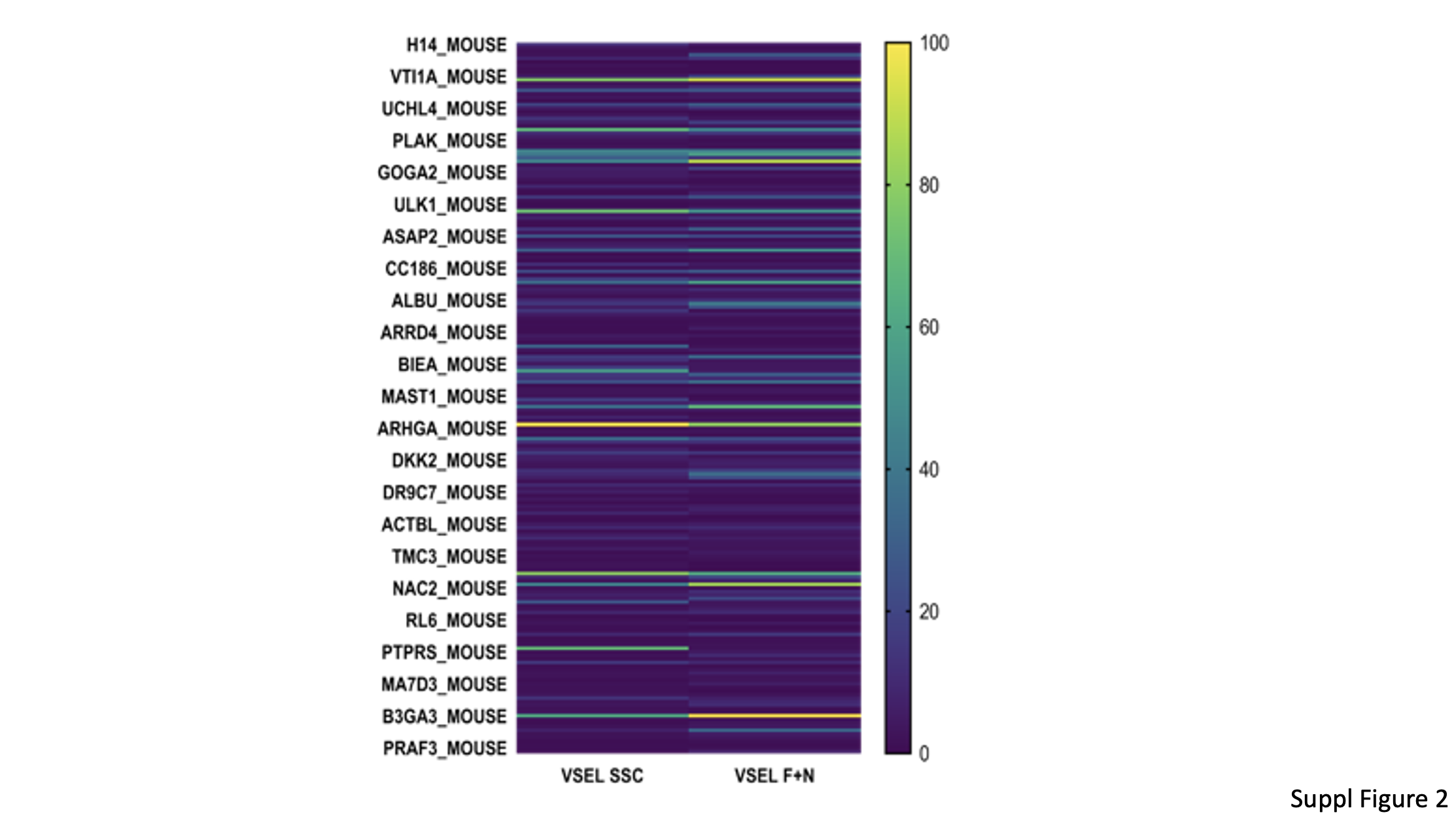

Supplement: Supplementary file 2 — High Resolution Image (TIFF 397 kb) [file 12015_2022_10445_MOESM2_ESM.tiff]
